# Analysis and Excavation of Unique Metabolic Components of Wheat Cultivated in Saline–Alkaline Soil

**DOI:** 10.3390/foods14223888

**Published:** 2025-11-13

**Authors:** Qiaozhi Song, Yu Liu, Ming Li, Lei Chang, Boli Guo

**Affiliations:** 1 Institute of Food Science and Technology, CAAS/Comprehensive Utilization Laboratory of Cereal and Oil Processing, Ministry of Agriculture and Rural, Beijing 100193, China; songqiaozhi@caas.cn (Q.S.); lemon_liuyu@126.com (Y.L.); minglicaas@126.com (M.L.); changcl775@163.com (L.C.); 2 Institute of Food Science Technology Nutrition and Health (Cangzhou) CAAS, Cangzhou 061019, China; 3 Zhongyuan Research Center, Chinese Academy of Agricultural Sciences, Xinxiang 453500, China

**Keywords:** wheat, metabolomics, differential metabolites, drought and saline–alkaline stress

## Abstract

In order to investigate the impact of drought and saline–alkaline stress on the growth and metabolic components of wheat, as well as to identify advantageous components of wheat under saline–alkaline conditions, metabolomics analysis was conducted separately on wheat cultivated in saline–alkaline soil at Zhong Jie Industrial Park (AAW) and generally grown wheat at Xian Huanyuan Village (GW). The results revealed that AAW exhibited higher levels of accumulated metabolites compared to GW. Specifically, under drought and saline–alkaline stress, alkaloids, flavones, amino acids, and derivatives were significantly up-regulated, while phenolic acids and terpenoids were down-regulated. Notably, 29 differential metabolites, including vitexin-2″-O-glucoside, N-feruloyl agmatine, apigenin-8-C-glucoside, and L-alanyl-L-phenylalanine, showed significant differences between AAW and GW. Flavone and flavonol biosynthesis, apigenin C-glycosides biosynthesis, and metabolic pathways were identified as key pathways contributing to the observed differences in metabolite production. Apigenin-8-C-glucoside and vitexin-2″-O-glucoside emerged as reliable biomarkers for distinguishing between AAW and GW. These findings suggest that metabolites unique to wheat grown in saline–alkaline soil may serve as biomarkers for developing stress-resistant varieties, warranting further study of their functional components in food products.

## 1. Introduction

Wheat (*Triticum aestivum* L.), an essential staple crop, is widely recognized for its yield and quality considerations in the context of environmental challenges, including global climate change, intermittent drought, and prolonged saline–alkaline stress [1]. Current agricultural research focuses on breeding drought-tolerant and alkali-tolerant wheat varieties, strategically utilizing saline–alkaline lands, and maximizing the potential of saline–alkaline land resources. Plants synthesize secondary metabolites (e.g., alkaloids, flavonoids, phenols) that are critical for stress responses and defense mechanisms [2,3]. These polyphenolic compounds exhibit diverse biological activities [4] (antioxidant, anti-inflammatory, etc.) and play key roles in plant adaptation to environmental stresses [5,6]. Quantifying their dynamic changes provides a powerful approach to deciphering plant physiological responses to stimuli.

Environmental stress can disrupt cell structures and impair essential physiological functions. Drought, salinity, and low temperature stress induce osmotic stress, leading to turgor loss and growth inhibition, thereby triggering plant stress response mechanisms. This can alter metabolic activities through oxidative stress, gene reprogramming, the activation of ion channels, and osmotic pressure synthesis to produce substances that stabilize cells [7,8]. Amino acids, organic acids, and sugars are among the primary metabolites affected by drought stress. Environmental conditions can promote the accumulation of specific metabolites, such as proline, a known osmotic stress marker in hard wheat [9]. Physiological and metabolic changes induced via drought stress can serve as indicators of plant sensitivity or tolerance to water deficit conditions [10]. Long-term soil drought can restrict pigment assimilation and the production of most free amino acids in wheat aboveground parts while promoting proline accumulation and the activation of chitinase and glucanase enzymes [11]. Additionally, alkaloids, organic acids, and flavonoids play significant roles in enhancing drought tolerance [12,13]. Soil salinity is also a severe abiotic stressor influencing plant growth [14]. Compatible solutes produced via salt-tolerant varieties or species under saline conditions provide beneficial effects on salt stress resistance [15,16]. Moreover, secondary metabolites such as phenolic acids, flavonoids, sugars, amino acids, and plant hormones are crucial for plant responses to salt stress [8]. For instance, under salinity stress, the biosynthesis pathways of phenylpropanoids, flavonoids, and lignin in *Setaria italica* roots play significant roles in response to salinity stress [17]. Salt-tolerant rice varieties exhibit increased levels of total phenols and flavonoids, vanillin, and protocatechuic acid under salt stress conditions [18]. Plants enhance tolerance to abiotic stresses, such as salt and osmotic stress, by increasing flavonoid accumulation in *Arabidopsis* [19]. The accumulation of phenols and flavonoids under salt stress aids plants in mitigating salt-induced oxidative stress [20]. These phenolic compounds possess antioxidant properties, while flavonoids exhibit anti-inflammatory, antiviral, anticancer, and anti-tumor activities [18,21,22,23] and also contribute to the taste of processed wheat products [24,25].

Metabolomics holds promise for addressing plant–environment interactions, yet most wheat metabolomics studies focus on grain composition or single-factor stress effects under controlled conditions, with limited research on plant responses to field environmental stress. Understanding the mechanisms of salinization challenges in global agriculture is essential. This study employs high-performance liquid chromatography–mass spectrometry (HPLC-MS) to analyze the metabolite differences in wheat subjected to drought–alkali stress in field experiments, aiming to identify key components and elucidate the impact of stress on wheat growth.

## 2. Materials and Methods

### 2.1. Wheat Materials

Three wheat (*Triticum aestivum* L.) varieties—Cangmai 6002, Cangmai 6005, and Jimai 44 (referred to as CM6002, CM6005, JM44)—were planted in the season of 2022–2023 in two experimental fields with different precipitation and salinity degrees in Cangzhou, Hebei Province. The study area is scientifically classified as a semi-arid dryland–meadow saline area within the Huang-Huai-Hai alluvial plain, one of the types of saline–alkaline lands in China. As a mild saline–alkaline soil with a pH of between 7.1 and 8.5, this type of land is more conducive to agricultural activities [26]. The specific information of the experimental fields is shown in Table 1. Zhong Jie Industrial Park has lower precipitation but higher pH and total salt content compared to Xian Huanyuan village. CM6002 and CM6005 are local winter wheat cultivars with drought resistance and salt tolerance traits. JM44 is a high-yielding semi-winter wheat variety with strong gluten properties. Each variety was planted in plots of one mu (1 mu = 666.667 m^2^), with five replicates per field arranged in a randomized complete block design. At maturity, wheat grain samples were harvested in the same season; from them, 5 kg portions were randomly extracted and labeled per sample. For analytical purposes, three subsamples (100 g each) were systematically drawn from the 5 kg batch per trial. A total of 18 wheat samples (2 fields × 3 genotypes × 3 replicates) were processed using an all-purpose disintegrator (ZK-100B, Zhongke Haoyu Technology Co., Ltd., Beijing, China). The fundamental nutritional and mineral contents in wheat grains from both soil conditions are shown in Tables S1 and S2, respectively.

### 2.2. Sample Preparation and Extraction

Wheat grain samples underwent freeze-drying in a Scientz-100F freeze-dryer before grinding for 1.5 min at 30 Hz using an MM 400 Retsch grinding machine. Each sample was subjected to three biological replicates for a widely targeted metabolomic analysis. After 50 mg of each sample was weighed, the samples were dissolved in methanol that had been pre-cooled to 70% at 20 °C at a ratio of 1200 μL: 50 mg. The dissolved samples were centrifuged at 12,000 rpm for 3 min, and the supernatant was filtered using a 0.22 μm-pore-size microporous membrane to obtain the sample extract. Quality control samples (QC) were created by mixing extracts from each sample; they were used to monitor the repeatability and reliability of the analysis process.

### 2.3. Analysis Conditions

The sample extracts were analyzed using the UPLC-ESI-MS/MS system (Ultra Performance Liquid Chromatography, UPLC, ExionLC™ AD; Tandem mass spectrometry, MS/MS; https://sciex.com.cn/).

The UPLC analysis conditions were as follows:

(1) Chromatographic column: Agilent SB-C18 (1.8 µm, 2.1 mm × 100 mm).
(2) Mobile phase: pure water with 0.1% formic acid (solvent A) and acetonitrile with 0.1% formic acid (solvent B).
(3) A gradient program was employed for sample measurement. The elution gradient proceeded as follows: initial condition—95% A, 5% B; a linear increase in B to 95% within 9 min; a decrease in A to 5%, maintained for 1 min; and an adjustment to 95% A and 5% B in 1.1 min, and then equilibrium for 14 min.
(4) During elution, the flow velocity was 0.35 mL/min; the column oven was set to 40 °C; the injection volume was 2 μL. The effluent was connected to an ESI-Q TRAP-MS/MS system.

The ESI-Q TRAP-MS/Ms conditions mainly included the following: electrospray ionization (ESI) in positive and negative ion modes. Source parameters: source temperature 550 °C; ion spray voltage 5500 V (positive ion mode)/−4500 V (negative ion mode); gas I (GSI), gas II (GSII), and curtain gas (CUR) at 50, 60, and 25 psi, respectively; and collision-activated dissociation (CAD) set to high.

Metabolite quantification utilized the multiple reaction monitoring (MRM) mode of triple quadrupole (QQQ) mass spectrometry, with nitrogen collision gas set to medium. The optimization of declustering potential and collision energy was performed for each MRM transition. Specific MRM ion pairs were monitored based on the elution time

### 2.4. Multivariate Statistical Analysis

Metabolite differences between AAW and GW were analyzed using principal component analysis (PCA), hierarchical cluster analysis (HCA), and orthogonal partial least squares–discriminant analysis (OPLS-DA). Metabolite data was normalized, log-transformed, and mean-centered before analysis. Unsupervised PCA was performed through the statistics function prcomp within R (www.r-project.org). ComplexHeatmap and MetaboAnalystR were conducted. For HCA, normalized signal intensities of metabolites (unit variance scaling) were visualized as a color spectrum. OPLS-DA was performed through 200 permutation tests to avoid overfitting. The OPLS-DA model was used to screen differential metabolites, and variable importance in the projection (VIP) ≥ 1 and absolute fold change (|Log_2_FC|) ≥ 1 were used as screening conditions. An S-plot diagram, a dynamic distribution map of the metabolite content difference, a volcano plot diagram, and a differential metabolite clustering heat map were generated using Metware Cloud, a free online platform for data analysis (https://cloud.metware.cn).

## 3. Results

### 3.1. Metabolic Profiling

**Figure 1:**
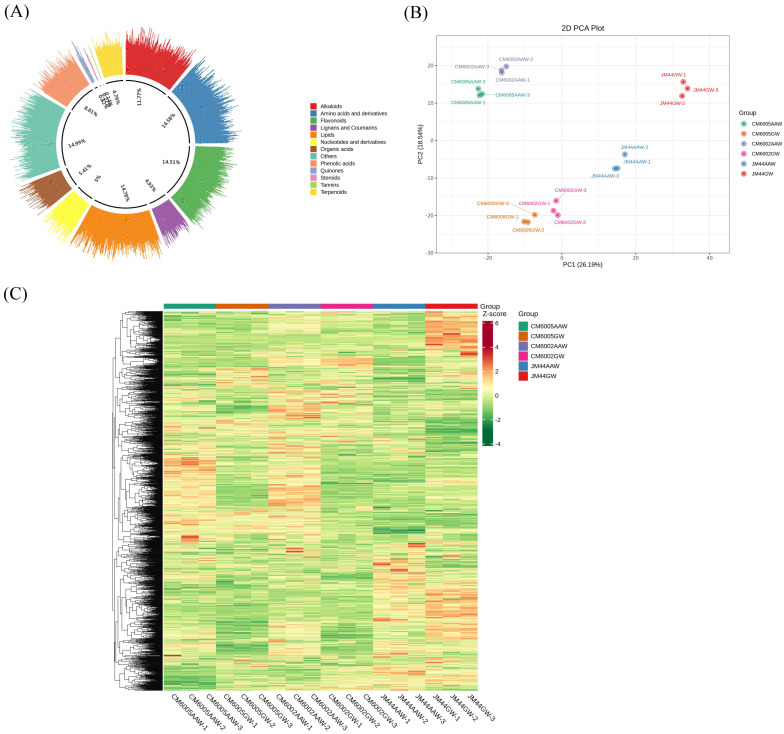
Sample grouping diagrams. (**A**) Metabolite classification proportion circle diagram; (**B**) grouped PCA chart; (**C**) overall cluster diagram of samples; note: the transverse is the name of the sample, while the longitudinal is the metabolite information. Different fill colors indicate different values obtained after the standardized treatment of different relative contents (red represents high content; green represents low content).

The metabolic profiles of six wheat samples—CM6002, CM6005, and JM44—grown in two different environments were analyzed using UPLC-MS/MS for targeted metabolites. A total of 1461 metabolites were detected across the samples using the UPLC-MS/MS detection platform and the self-built MWDB database (Metware Database) (Table S3). These metabolites were categorized into 13 main groups based on their chemical properties and structures (Figure 1A), including lipids (14.78%), amino acids and derivatives (14.58%), flavonoids (14.51%), alkaloids (11.77%), phenolic acids (8.01%), organic acids (5.41%), nucleotides and derivatives (5%), lignans and coumarins (4.93%), terpenoids (4.79%), quinones (0.82%), steroids (0.27%), tannins (0.14%), and others (14.99%).

### 3.2. Multivariate Statistical Analysis

To investigate metabolite differences among the wheat samples, a multivariate statistical analysis was conducted. The unsupervised PCA model was utilized to evaluate overall variations between the six samples. Different-colored and -shaped points exhibited varying degrees of clustering or separation in a two-dimensional space, indicating metabolic distinctions between groups and samples within groups. The PCA score plot revealed that the two principal components explained 48.1% of the total variance, with PC1 contributing 32.48% and PC2 contributing 28.09% of the variance, respectively. Figure 1B demonstrates distinct clustering patterns among sample groups, with a close clustering of replicates within each group, confirming the reproducibility and reliability of the detection methods. Notably, significant separation between sample groups suggested substantial variations in metabolic profiles. Hierarchical Cluster Analysis (HCA) highlighted three main accumulation clusters of metabolites across samples (Figure 1C). Cluster 1 displayed higher metabolite accumulation levels in GW varieties, while cluster 2 showed elevated accumulation levels in AAW samples of CM6002 and CM6005, and cluster 3 exhibited higher accumulation levels in JM44. These findings underscore significant metabolic differences between GW and AAW samples, with AAW exhibiting higher metabolite accumulation levels compared to GW.

### 3.3. Identification of Differential Metabolites

**Figure 2:**
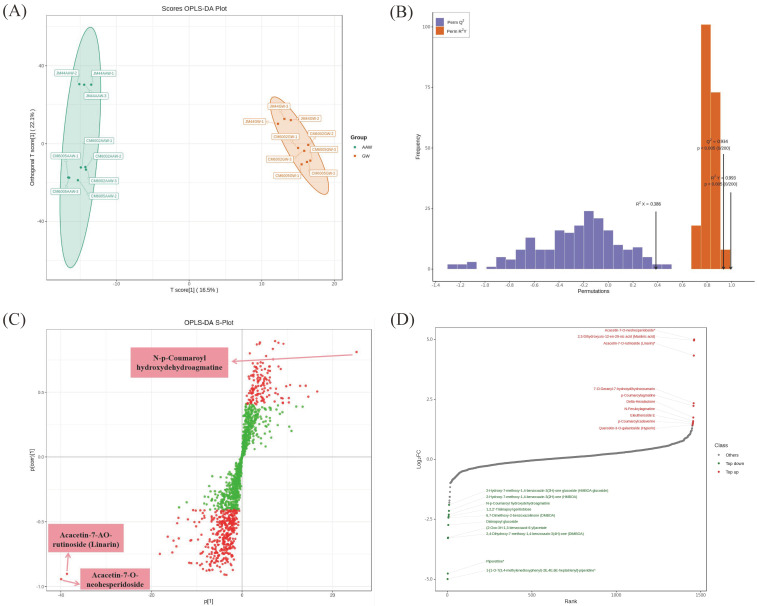
(**A**) OPLS-DA score plot. (**B**) OPLS-DA verification diagram. (**C**) OPLS-DA S-plot in which the abscissa represents the covariance between the principal component and the metabolite, and the ordinate represents the correlation coefficient between the principal component and the metabolite. The red point indicates that the VIP value of these metabolites is greater than 1, and the green point indicates that the VIP value of these metabolites is less than or equal to 1. (**D**) Dynamic distribution map of metabolite content difference; note: in the figure, the abscissa represents the cumulative number of substances arranged from small to large according to the difference multiple, the ordinate represents the logarithm of the difference multiple based on 2, the green point represents the top 10 substances that are down-regulated, and the red point represents the top 10 substances that are up-regulated.

OPLS-DA, a supervised multivariate statistical analysis method for pattern recognition, was utilized to differentiate between groups and identify distinctive metabolites by eliminating unrelated orthogonal variables. OPLS-DA modeling was performed on GW and AAW samples of the three wheat varieties using the OPLSR (Figure 2A) and R package MetaboAnalystR 4.0. The metabolomics data were analyzed using the OPLS-DA model, and score plots were generated for each group to visualize inter-group differences. The Scores OPLS-DA Plot revealed minor differences between the wheat varieties, with clear separation between AAW and GW, highlighting significant discrepancies identified via the OPLS-DA model. To validate the model’s reliability, 200 random permutation tests were conducted, showing R^2^Y and Q^2^ scores exceeding 0.9 with *p* < 0.005 (Figure 2B), indicating the model’s stability and predictive capability for screening differential metabolites.

The S-plot of OPLS-DA (Figure 2C) identified metabolites with a VIP ≥ 1 between AAW and GW. Metabolites with VIP values above 1 (red dots) were considered significant, with a total of 523 metabolites, while those below or equal to 1 (green dots) were less relevant. Metabolites closer to the upper right and lower left corners indicated greater differences, such as N-p-Coumaroyl hydroxydehydroagmatine, acacetin-7-O-neohesperidoside, and acacetin-7-O-rutinoside (linarin), which were categorized as alkaloids and flavonoids, respectively.

For a comprehensive overview of metabolic distinctions, FC values were calculated for metabolites in the comparison group. The top 10 up-regulated and down-regulated metabolites were labeled based on FC values (Figure 2D), highlighting differences in flavonols, phenolamine, phenolic acids, and lignans between AAW and GW.

**Figure 3:**
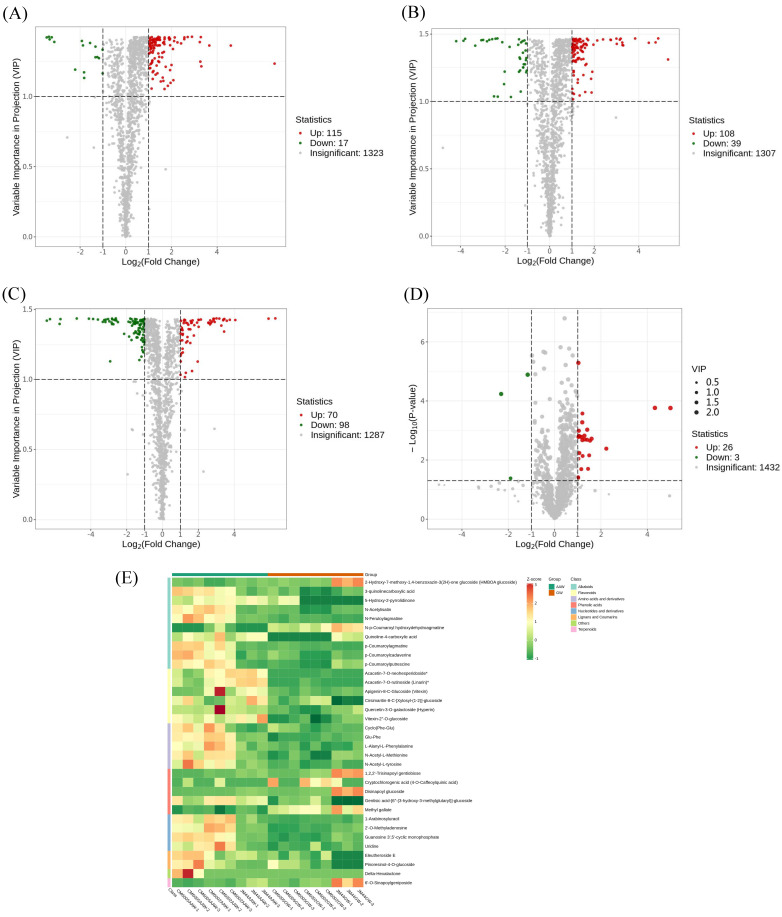
(**A**–**D**) Volcano plots of differential metabolites of CM6002, CM6005, JM44, and total samples, AAW vs. GW; note: the green point represents a down-regulated differential metabolite, the red point represents an up-regulated differential metabolite, and the gray represents a metabolite that is detected but not significantly different. (**E**) Cluster heat map of differential metabolites. (Note: AAW—wheat cultivated in saline–alkaline soil at Zhong Jie Industrial Park; GW—generally grown wheat at Xian Huanyuan Village)

The differential metabolites were further refined; those with a VIP > 1 and an FC ≥ 2 or an FC ≤ 0.5 were selected. Volcano plots (Figure 3A–C) depicted 132 differential metabolites (115 up-regulated, 17 down-regulated) between AAW and GW of CM6002, 147 metabolites (108 up-regulated, 39 down-regulated) between AAW and GW of CM6005, and 168 significant metabolites (70 up-regulated, 98 down-regulated) between AAW and GW of JM44. The volcano plots and cluster heatmap of differential metabolites for all samples (AAW vs. GW) were presented in Figure 3D and Figure 3E, respectively. Thirty-four metabolites displayed significant differences between AAW and GW, as shown in Table 2, including 27 up-regulated metabolites, such as uridine, eleutheroside E, apigenin-8-C-glucoside (vitexin), vitexin-2”-O-glucoside, cyclo (Phe-Glu), and p-coumaroylagmatine, and 7 down-regulated metabolites, including 2-hydroxy-7-methoxy-1,4-benzoxazin-3(2H)-one glucoside (HMBOA glucoside), N-p-coumaroyl hydroxydehydroagmatine, 6′-O-sinapoylgeniposide, 1,2,2′-trisinapoyl gentiobiose, cryptochlorogenic acid (4-O-caffeoylquinic acid), disinapoyl glucoside, and methyl gallateg.

Furthermore, through the imposition of additional criteria (*p*-value < 0.05), five metabolites were excluded, including delta-hexalactone, disinapoyl glucoside, 6′-O-sinapoylgeniposide, 1,2,2′-trisinapoyl gentiobiose, and cryptochlorogenic acid (4-O-caffeoylquinic acid), leading to 29 differential metabolites (26 up-regulated, 3 down-regulated), mainly comprising secondary metabolites between AAW and GW. The up-regulated metabolites were predominantly alkaloids, flavonoids, amino acids, and derivatives, while down-regulated metabolites primarily included phenolic acids and a small number of alkaloids. These results were consistent with the above analysis and served as representative differential metabolites between AAW and GW.

### 3.4. KEGG Annotation and Enrichment Analysis of Differential Metabolites

Metabolites interact within organisms to create various pathways. The KEGG (Kyoto Encyclopedia of Genes and Genomes) database is a primary public resource for pathways, offering comprehensive metabolic pathway information essential for in vivo metabolic analysis and network research. In this study, we utilized the KEGG database to annotate and enrich the differential metabolites present in both AAW and GW. The annotations for these differential metabolites can be found in Table S4.

**Figure 4:**
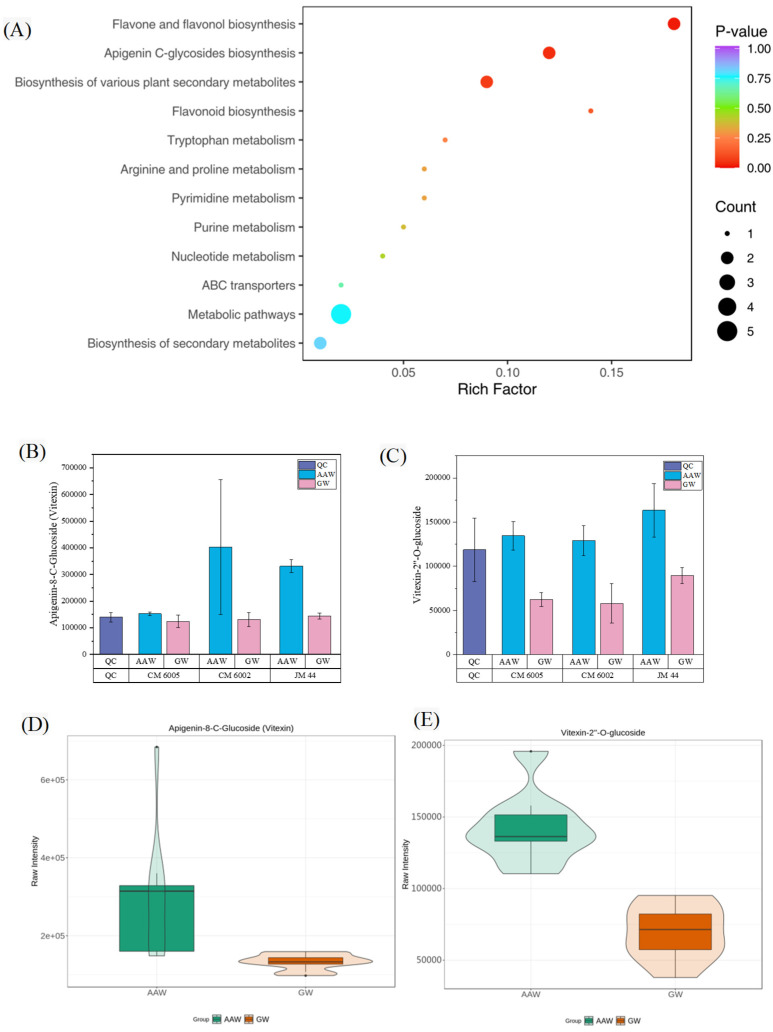
(**A**) AAW vs. GW differential metabolite pathway enrichment map; note: the abscissa represents the rich factor corresponding to each pathway, and the ordinate is the pathway name (sorted by *p*-value). The color of the point reflects the size of the *p*-value, and red indicates that the enrichment is more significant. The size of the point represents the number of differential metabolites enriched. (**B**,**C**) The relative content of apigenin-8-C-glucoside (vitexin) and vitexin-2′-O-glucoside in AAW and GW. (**D**,**E**) Violin maps of the differential metabolites apigenin-8-C-glucoside (vitexin) and vitexin-2′-O-glucoside in AAW and GW. (Note: AAW—wheat cultivated in saline–alkaline soil at Zhong Jie Industrial Park; GW—generally grown wheat at Xian Huanyuan Village)

Our analysis revealed that the differential metabolites were predominantly enriched in 12 metabolic pathways, including flavonoid biosynthesis, flavone and flavonol biosynthesis, apigenin C-glycosides biosynthesis, the biosynthesis of secondary metabolites, the biosynthesis of various plant secondary metabolites, purine metabolism, tryptophan metabolism, pyrimidine metabolism, nucleotide metabolism, arginine and proline metabolism, and ABC transporters. Within the metabolic pathways, five significantly different metabolites were identified, namely guanosine 3′,5′-cyclic monophosphate, N-feruloylagmatine, uridine, p-coumaroylagmatine, and p-coumaroylputrescine (Figure 4A). These metabolites primarily belong to the categories of chenolamine (alkaloids) and nucleotides and derivatives. Furthermore, our analysis revealed that two differential metabolites were annotated to flavone and flavonol biosynthesis, apigenin C-glycosides biosynthesis, the biosynthesis of various plant secondary metabolites, and the biosynthesis of secondary metabolites. These metabolites consist of flavones (flavonoids) and phenolamine (alkaloids) compounds.

**Figure 5:**
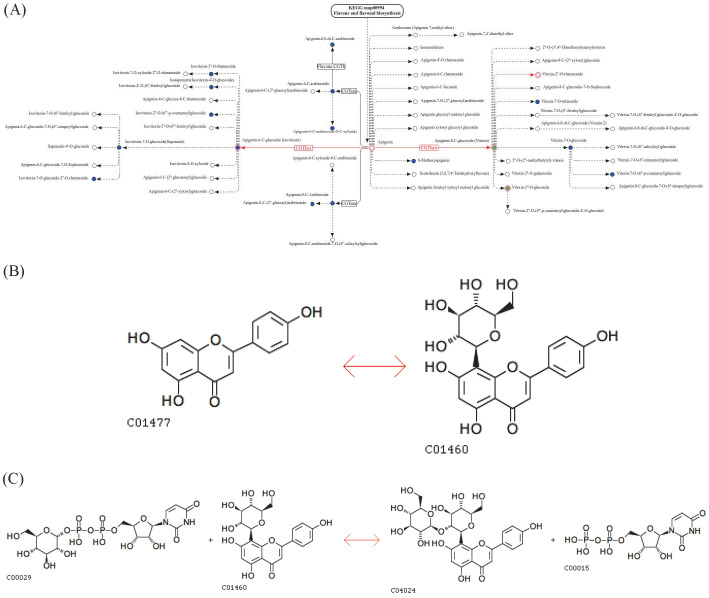
Pathway analysis diagram of iconic differential metabolites. (**A**–**C**) Flavone and flavonol biosynthesis metabolic pathway map and synthesis pathway; note: red indicates that the metabolite content was significantly down-regulated in the experimental group, blue indicates that the metabolite was detected but did not change significantly, and green indicates that the metabolite content was significantly up-regulated in the experimental group. C01477: Apigenin; C00029: UDP-glucose; C01460: Apigenin-8-C-glucoside (vitexin); C04024: Vitexin-2”-O-glucoside; C00015: Uridine 5′-diphosphate (UDP).

The comparison of these metabolites across three wheat varieties showed consistent differences (Figure 4B,C), with AAW exhibiting higher relative content (Figure 4D,E). Notably, the KEGG pathway analysis of flavone and flavonol biosynthesis indicated that apigenin serves as the main precursor to apigenin-8-C-glucoside (vitexin), which can further be converted into vitexin-2”-O-glucoside via a hexosyltransferase reaction (Figure 5A–C). This pathway may contribute to the unique metabolic profile observed in AAW.

These results indicated that the metabolite profiles of wheat cultivated in different environments vary significantly, with flavone and flavonol biosynthesis, apigenin C-glycosides biosynthesis, and metabolic pathways playing crucial roles in driving these differences. Alkaloids, flavonoids, and nucleotides and their derivatives are identified as key contributors to the divergence between AAW and GW, with apigenin-8-C-glucoside (vitexin) and vitexin-2”-O-glucoside emerging as reliable biomarkers distinguishing the metabolites between the wheat grown under two soil conditions.

## 4. Discussion

Plants are exposed to various environmental stresses, such as drought, alkalinity, salinity, extreme temperatures, heavy metals, and pesticides [27], leading to oxidative stress and reduced crop yields [28] and posing a threat to food security. To combat these stresses, plants have developed protective mechanisms.

### 4.1. Differential Metabolites of AAW and GW

In this study, a wide range of targeted metabolites were analyzed in wheat grains planted in drought and saline–alkaline stress soil environments, as well as common land. It was found that lipids, amino acids, and derivatives were the main primary metabolites, while alkaloids and flavonoids were the main secondary metabolites. Changes in primary metabolism, particularly nitrogen-transporting and aromatic amino acids, can enhance a plant’s resistance to adverse environments [29]. Furthermore, under environmental stress conditions, plant lipid composition and molecular levels will also change due to the activation of various enzymes [30]. Secondary metabolites such as flavonoids and alkaloids are mainly involved in the activation of plant signal transduction and plant defense mechanisms [6]. The accumulation of these substances can enhance the tolerance of plants to stress [8].

The differences in metabolites observed in six wheat samples were influenced by genotype and planting environment. Studies have shown that the content of some nucleotides in drought-tolerant-genotype wheat was significantly higher than that in drought-sensitive-genotype wheat [12]. Variations in phytosterol content and tocopherol accumulation were also noted among different wheat varieties and in response to rainfall levels [9]. Factors such as wheat genotype, growth environment, and soil conditions impact lipid content, category, and fatty acid levels in wheat grains [31].

The differential metabolites of AAW and GW were mainly secondary metabolites such as alkaloids and flavonoids, followed by primary metabolites such as amino acids and nucleotides. Studies have shown that the content of some nucleotides in drought-tolerant genotypes of wheat is significantly higher than that in drought-sensitive genotypes, and thymine and guanine may promote drought tolerance by generating energy and enhancing protective responses. In addition, some high levels of amino acids, alkaloids, organic acids, and flavonoid metabolites in drought-treated wheat may help explain its strong drought tolerance. In general, plants with poor drought tolerance are more susceptible to drought stress at both the transcriptional and metabolic levels, due to the lack of a steady-state mechanism to mitigate the effects of water deficit, and drought-responsive genes related to stress response, reactive oxygen species scavenging, etc., are more likely to be activated [32], thereby affecting the enrichment level of metabolites. Flavonoids play an important role in drought tolerance. Lv et al. found that the improvement in antioxidant enzyme activity and the increase in flavonoids led to an increase in amino acid biosynthesis and ROS scavenging ability [13]. Flavonoids such as apigenin-3-O-rhamnoside and chrysophanol-O-malonylhexoside contributed more to non-enzymatic antioxidants. This study found that flavonoids or flavonols such as vitexin-2”-O-glucoside, apigenin-8-C-glucoside (vitexin), and quercetin-3-O-galactoside (hyperin) were significantly up-regulated in AAW, which indicated that wheat grown in stressful environments exhibits more effective antioxidant defense capabilities to cope with ROS-induced oxidative stress.

### 4.2. Effects of Drought and Saline–Alkaline Stress on Wheat Metabolites

Abiotic stresses, such as drought and saline–alkaline conditions, have profound impacts on plant metabolism. The effects of drought stress mainly depend on factors such as genotype, stress degree, stress mode, stress duration, and climatic conditions [11,12,33,34,35]. It has been reported that metabolites such as amino acids, organic acids, nucleosides, alkaloids, flavonoids, and phenolic compounds in wheat increased significantly under drought stress conditions, while shikimic acid and ferulic acid decreased under drought stress conditions [35]. Consistent with these studies, our research identified a significant up-regulation of seven alkaloids, including phenolamines, plumeranes, and pyridines, in AWW among the major differential metabolites. Additionally, compared to GW, AAW grains showed significant up-regulation in five amino acids, such as L-alanyl-L-phenylalanine and N-acetyl-L-methionine, four nucleotides, including 2′-O-methyladenosine and uridine, as well as six flavonoids, namely quercetin-3-O-galactoside (hyperin) and cirsimaritin-8-C-[xylosyl-(1-2)]-glucoside. The accumulation of these metabolites enhances the stress response in wheat during growth and development processes in the face of adversity.

Under salt stress, crop growth, the yield potential, and grain quality may be negatively affected [36]. There were also complex interactions between internal physiological and biochemical processes [18]. Salinization can change the accumulation of secondary metabolites in rosemary, especially the composition of monoterpenes [22]. Under arsenate stress, several metabolites involved in the amino acid biosynthesis pathway in wheat will be regulated and significantly changed to promote the defense response under stress [36]. The saline–alkaline planting environment will change the metabolism of plants, which may be due to reactive oxygen species (ROS) produced in large quantities caused by oxidation. To cope with abiotic stress, plants also produce a large number of secondary metabolites to scavenge or detoxify ROS. For example, phenols and flavonoids in plants may help plants reduce salt-induced oxidative stress and accumulate in large quantities under salt stress conditions [20]. The contents of total phenolics, vanillin, and protocatechuic acid in salt-tolerant rice also increased significantly under salt stress [18].

In addition, salinity affects ion composition and the osmotic balance in plant cells. Environmental stresses such as drought and saline–alkaline stress can produce osmotic stress, which destroys the cell structure of plants, leads to expansion loss, and damages key physiological functions [37]. Osmotic regulation is crucial for plants to manage external stresses, uphold cell hydration, and restore osmotic equilibrium [38]. Key osmolytes, such as amino acids (especially proline), glycine betaine, soluble sugars, proteins, polyamines, and organic acids, accumulate under various stresses [27], bolstering cell tolerance without interfering with plant-cell mechanisms and stabilizing membranes, protein, and other subcellular structures under osmotic stress. Our study revealed the significant up-regulation of phenolic amines such as p-coumaroylputrescine, p-coumaroylcadaverine, p-coumaroylagmatine, N-feruloylagmatine, and amino acids and their derivatives, such as Glu-Phe, N-acetyl-L-methionine, L-alanyl-L-phenylalanine, N-acetyl-L-tyrosine, and cyclo (Phe-Glu) in AAW under abiotic stresses such as drought and saline–alkaline stress, indicating adjustments in organic metabolite levels in wheat to regulate the osmotic balance and counter oxidative damage.

Soil salinization is considered a major environmental threat to agricultural systems; additionally, alkaline stress exhibits similar stress factors to salt stress and may have synergistic effects [39]. Salt and alkaline stress primarily induces osmotic stress and ion damage by disrupting ion homeostasis and balance in plant cells. High pH levels lead to proton deficiency, and they may even disrupt or inhibit the transmembrane electrochemical potential gradient in cells [40]. Therefore, plants actively adjust their metabolism to alleviate cellular damage and cope with abiotic stress. For example, wheat plants regulate pH in response to alkali stress by secreting various metabolites containing -COOH groups from their roots. Moreover, during the response process to alkali stress, wheat enhances the accumulation of metabolites, and enhanced glycolysis, fatty acid synthesis, and phenolic acid synthesis provide roots with more energy and substrates for secretion [39]. During the root or seedling stage, energy and high levels of organic acids may be key adaptive mechanisms for wheat to maintain the ion balance within cells under environmental stresses such as saline–alkaline stress. However, research by Guo et al. found that, under alkali stress, levels of certain amino acids and sugars in wheat increase, including the levels of proline, arginine, sucrose, sorbitol, trehalose, soluble sugars, and gentiobiose [40]. This may be due to the fact that, in mature grains and fruits, plants allocate resources towards storing energy and nutrients to meet growth and development needs. Consequently, levels of phenolic acids and organic acids in grains may decrease to increase the content of proteins and carbohydrates. This study found that wheat grains grown under drought and saline–alkaline stress conditions exhibited alterations in annotated metabolic pathways related to amino acids. Specifically, N-acetyl-L-tyrosine, L-alanyl-L-phenylalanine, N-acetyl-L-methionine, Glu-Phe, and other branched-chain amino acids (BCAAs) were significantly up-regulated. The accumulation of BCAAs is often a result of protein degradation and the activation of synthetic pathways [41], which may contribute to maintaining cellular function and the nutrient balance, thereby enhancing wheat plants’ adaptive capacity to drought and saline–alkaline stress. Therefore, changes in the levels of both primary and secondary metabolites play crucial physiological roles in plant responses to environmental stress, providing an important regulatory mechanism for wheat-plant survival and growth.

### 4.3. Specific Metabolic Changes Under Drought and Saline–Alkaline Stress Conditions

Specific changes in metabolite levels may arise from the inhibition or the activation of specific metabolic pathways. Therefore, these metabolites may serve as ideal targets for studying the effects of specific abiotic stress and adaptation mechanisms [41]. In this study, a total of 29 metabolites exhibited specific changes in AAW compared to GW. Among these metabolites, 26 were significantly up-regulated, with 8 differential metabolites annotated to 12 metabolic pathways in the KEGG database. These pathways include four nucleotide-related metabolic pathways, three flavonoid biosynthesis pathways, two amino acid metabolic pathways, and three secondary metabolic pathways. Notably, pathways related to flavone and flavonol biosynthesis and apigenin C-glycosides biosynthesis showed significant enrichment in the KEGG pathways.

Lipids and proteins are the primary target sites for oxidative stress in plants exposed to abiotic stress [42]. Unfavorable environmental conditions can directly or indirectly cause serious damage to plants, trigger ROS production, and form lipid or nucleotide peroxides, causing oxidative stress-induced lipid peroxidation, membrane system impairment, and cellular damage [43]. The regulation of key enzyme activities, non-enzymatic systems, and signal transduction pathways is essential in response to oxidative stress under this abiotic stress. Among non-enzymatic substances, flavonoids were considered to exhibit an excellent ROS scavenging ability [44] and play an important role in response to drought and saline–alkaline stress. Our results highlighted that the KEGG pathways enriched through differential metabolites between AAW and GW were mainly flavonoid, flavone, and flavonol metabolic pathways. Under drought and saline–alkaline conditions, the content of flavonoids in wheat increased, which aligned with previous studies [45]. Flavonoid accumulation was facilitated via the up-regulation of key enzyme-coding genes, such as chalcone synthase (CHS) and chalcone isomerase (CHI), in biosynthesis pathways under stress conditions [46].

In the results, vitexin and its derivatives emerged as prominent differential flavonoid metabolites. Vitexin, a kind of apigenin flavonoid glycoside and C-glycosylated flavonoid, exhibits pharmacological diversity [47]. Studies have shown that vitexin and its derivatives demonstrate anti-fat formation properties and various medicinal benefits, such as antiviral, antibacterial, antioxidant, anti-inflammatory, anti-tumor, and hypotensive effects [48,49], which were primarily sourced from medicinal plants, such as mimosa, passionfruit, and chayote. Vitexin showcases superior antioxidant activity compared to apigenin due to its C-8 glucoside structure, enabling effective free-radical scavenging [50]. Higher levels of vitexin were observed in AAW compared to GW, especially apigenin-8-C-glucoside (vitexin) and vitexin-2”-O-glucoside. This is a very interesting finding: vitexin, as an antioxidant, has a protective effect on ROS, lipid peroxidation, and other oxidative damage, but it has a relatively high content in the extensive food source of AAW. This finding paves the way for the utilization of vitexin as a promising target for natural antioxidant development, offering new insights and research directions for efficient saline–alkali utilization, the optimal quality mining of AAW, and strategic support for the AAW industry.

## 5. Conclusions

The differential metabolic profiles between wheat grown under two soil conditions revealed in this study highlight the potential of wheat under saline–alkaline conditions as a functional food ingredient. Under combined drought and saline–alkaline stress, AAW exhibited the significant up-regulation of 27 metabolites, including bioactive compounds, such as asuridine, eleutheroside E, apigenin-8-C-glucoside (vitexin), vitexin-2”-O-glucoside, cyclo (Phe-Glu), and p-coumaroylagmatine. Conversely, the relative content of seven metabolites, such as N-p-coumaroyl hydroxydehydroagmatine and 6′-O-sinapoylgeniposide, was lower. Overall, some alkaloids, flavones, amino acids and derivatives, nucleotides and derivatives, lignans, and coumarins were significantly up-regulated, while some phenolic acids and terpenoids were down-regulated. A differential metabolite analysis, including OPLS-DA, identified 29 key metabolites, such as vitexin-2”-O-glucoside, N-feruloylagmatineh, apigenin-8-C-glucoside (vitexin), and L-alanyl-L-phenylalanine, which distinguished AAW from GW. The KEGG pathway enrichment analysis suggested that flavone and flavonol biosynthesis, apigenin C-glycosides biosynthesis, and metabolic pathways were the main drivers of these differences. These pathways are directly linked to the production of bioactive compounds with relevance to cereal science, including potential applications in the flavor enhancement and health benefit optimization of wheat-based food. Notably, apigenin-8-C-glucoside (vitexin) and vitexin-2”-O-glucoside emerged as reliable biomarkers for distinguishing metabolite profiles between the wheat grown under two soil conditions. This study provides a theoretical foundation for the rational utilization of saline–alkaline wheat in food systems. In summary, our findings not only advance the understanding of wheat stress adaptation but also open new avenues for the food industry to harness the metabolic advantages of AAW, aligning with global trends toward nutrient-dense wheat-based food ingredients.

## Figures and Tables

**Table 1:** The soil information of the wheat-planting area.

Planting Site	Altitudes [m]	Precipitation [mm]	Soil Horizon [cm]	pH (Mean ± s.d.)	Total Salt Content (Mean ± s.d.) [g/kg]
Xian Huanyuan village	8	735.1	0–60	7.77 ± 0.20	1.37 ± 0.21
Zhong Jie industrial park	2.5	689.4	0–60	8.26 ± 0.32	2.70 ± 1.86

Note: Soil information for the sampling sites was provided by the Academy of Agriculture and Forestry Sciences, Hebei Key Laboratory of Drought-Alkali Tolerance in Wheat, Cangzhou, China.

**Table 2:** List of differential metabolites, AAW vs. GW.

Kernel	Compounds	CAS	Class I	Class II	Type
1	2-Hydroxy-7-methoxy-1,4-benzoxazin-3(2H)-one glucoside (HMBOA glucoside)	17622-26-3	Alkaloids	Alkaloids	down
2	3-quinolinecarboxylic acid	-	Alkaloids	Quinoline alkaloids	up
3	5-Hydroxy-2-pyrrolidinone	62312-55-4	Alkaloids	Pyrrole alkaloids	up
4	N-Acetylisatin	574-17-4	Alkaloids	Plumerane	up
5	N-Feruloylagmatine	-	Alkaloids	Phenolamine	up
6	N-p-Coumaroyl hydroxydehydroagmatine	-	Alkaloids	Phenolamine	down
7	Quinoline-4-carboxylic acid	486-74-8	Alkaloids	Quinoline alkaloids	up
8	p-Coumaroylagmatine	7295-86-5	Alkaloids	Phenolamine	up
9	p-Coumaroylcadaverine	-	Alkaloids	Phenolamine	up
10	p-Coumaroylputrescine	34136-53-3	Alkaloids	Phenolamine	up
11	Cyclo(Phe-Glu)	-	Amino acids and derivatives	Amino acids and derivatives	up
12	Glu-Phe	20556-22-3	Amino acids and derivatives	Amino acids and derivatives	up
13	L-Alanyl-L-Phenylalanine	3061-90-3	Amino acids and derivatives	Amino acids and derivatives	up
14	N-Acetyl-L-Methionine	65-82-7	Amino acids and derivatives	Amino acids and derivatives	up
15	N-Acetyl-L-tyrosine	537-55-3	Amino acids and derivatives	Amino acids and derivatives	up
16	Acacetin-7-O-neohesperidoside	20633-93-6	Flavonoids	Flavones	up
17	Acacetin-7-O-rutinoside (Linarin)	480-36-4	Flavonoids	Flavones	up
18	Apigenin-8-C-Glucoside (Vitexin)	3681-93-4	Flavonoids	Flavones	up
19	Cirsimaritin-8-C-[Xylosyl-(1-2)]-glucoside	-	Flavonoids	Flavones	up
20	Quercetin-3-O-galactoside (Hyperin)	482-36-0	Flavonoids	Flavonols	up
21	Vitexin-2″-O-glucoside	61360-94-9	Flavonoids	Flavones	up
22	Eleutheroside E	39432-56-9	Lignans and Coumarins	Lignans	up
23	Pinoresinol-4-O-glucoside	41607-20-9	Lignans and Coumarins	Lignans	up
24	1-Arabinosyluracil	3083-77-0	Nucleotides and derivatives	Nucleotides and derivatives	up
25	2′-O-Methyladenosine	2140-79-6	Nucleotides and derivatives	Nucleotides and derivatives	up
26	Guanosine 3′,5′-cyclic monophosphate	7665-99-8	Nucleotides and derivatives	Nucleotides and derivatives	up
27	Uridine	58-96-8	Nucleotides and derivatives	Nucleotides and derivatives	up
28	Delta-Hexalactone	823-22-3	Others	Lactones	up
29	1,2,2′-Trisinapoyl gentiobiose	-	Phenolic acids	Phenolic acids	down
30	Cryptochlorogenic acid (4-O-Caffeoylquinic acid)	905-99-7	Phenolic acids	Phenolic acids	down
31	Disinapoyl glucoside	-	Phenolic acids	Phenolic acids	down
32	Gentisic acid-[6″-(3-hydroxy-3-methylglutaryl)]-glucoside	-	Phenolic acids	Phenolic acids	up
33	Methyl gallate	99-24-1	Phenolic acids	Phenolic acids	down
34	6′-O-Sinapoylgeniposide	1012306-66-9	Terpenoids	Monoterpenoids	down

Note: “up” indicates that the differential metabolite is up-regulated; that is, the relative content is high in AAW and low in GW. “Down” indicates that the differential metabolite is down-regulated; that is, the relative content is low in AAW and high in GW. (Note: AAW—wheat cultivated in saline–alkaline soil at Zhong Jie Industrial Park; GW—generally grown wheat at Xian Huanyuan Village)

## Data Availability

The original contributions presented in this study are included in the article/Supplementary Materials. Further inquiries can be directed to the corresponding author.

## Supplementary Materials

The following supporting information can be downloaded at https://www.mdpi.com/article/10.3390/foods14223888/s1, Table S1: Comparison of nutritional substances between AAW and GW; Table S2: Comparison of mineral contents between AAW and GW; Table S3: All detected metabolites and their relative contents in wheat samples; Table S4: Differential metabolites and KEGG annotations in AAW and GW.

## Abbreviations

The following abbreviations are used in this manuscript:

AAW	Wheat cultivated in saline–alkaline soil at Zhong Jie Industrial Park
GW	Generally grown wheat at Xian Huanyuan Village
PCA	Principal component analysis
HCA	Hierarchical cluster analysis
OPLS-DA	Orthogonal partial least squares–discriminant analysis
KEGG	Kyoto Encyclopedia of Genes and Genomes
