# Supplementary material for: Analysis and Excavation of Unique Metabolic Components of Wheat Cultivated in Saline–Alkaline Soil
Source: Foods. 2025 Nov 13;14(22):3888. doi: 10.3390/foods14223888 (PMC12651151; doi:10.3390/foods14223888)
Supplement: Supplementary file 1 [file foods-14-03888-s001.zip › Supplementary Materials.pdf]

Table S1. Comparison of Nutritional Substances Between AAW and GW

Nutritional Substance	AAW	GW
Moisture (%)	13.21±0.72a	13.88±0.37b
Protein (%)	13.52±0.69a	11.68±0.71b
Amylose (dry basis, %)	24.46±0.67a	23.51±0.46b
Dietary Fiber (%)	18.94±1.75a	18.30±1.64b

Note: Different lowercase letters in the same row indicate significant differences ($P < 0.05$).

Table S2. Comparison of Mineral Contents Between AAW and GW

Mineral Content (mg/kg)	AAW	GW
K	3580.78±589.7a	3563.92±446.7a
Ca	358.64±116.96b	386.78±89.69a
Na	65.86±18.8a	30.03±9.53b
Mg	1216.43±252.14a	1223.25±228.1a
P	2826.78±658.77b	3138.98±772.16a
Fe	74.22±21.44a	66.64±17.96b
Zn	37.81±0.17a	32.37±1.52b
Cu	7.46±2.58b	8.21±3.92a
Mn	36.54±9.25a	38.33±5.43a
Sn	1.67±1.35a	1.47±1.21a

Note: Different lowercase letters in the same row indicate significant differences ($P < 0.05$).
